# Pulmonary Tuberculosis With Tuberculous Right Wrist Tenosynovitis, Hepatitis C, and Mild COVID-19 in a Kidney Transplant Indian Male: World’s First Case

**DOI:** 10.7759/cureus.28847

**Published:** 2022-09-06

**Authors:** Sankalp Yadav

**Affiliations:** 1 Medicine, Shri Madan Lal Khurana Chest Clinic, Moti Nagar, New Delhi, IND

**Keywords:** kidney transplant recipients, renal transplant, infectious tenosynovitis, hepatitis c (hcv) infection, sars-cov-2 (severe acute respiratory syndrome coronavirus -2), tuberculosis

## Abstract

The coronavirus disease 2019 (COVID-19) pandemic has resulted in large-scale devastation. Reports of COVID-19 in patients with compromised immunity are available in the literature. The compromised immunity could be due to multiple factors like drug induced as in organ transplant patients, diabetes, HIV, etc. Post-transplant patients with compromised immunity due to immune suppression are vulnerable to many infections (tuberculosis, hepatitis B and C, etc.). Herein a case of an Indian male with a kidney transplant is presented who had concurrent infections of pulmonary and extrapulmonary tuberculosis due to *Mycobacterium tuberculosis*, hepatitis C virus, and severe acute respiratory syndrome coronavirus 2 (SARS‑CoV‑2). A detailed history with laboratory workup was done to establish the diagnosis and a prompt treatment was initiated for the three infections. To the best knowledge of the author, no such case has ever been reported in the medical literature to date. The management of this rare case is highlighted in this present write-up.

## Introduction

The pandemic of coronavirus disease 2019 (COVID-19) caused by severe acute respiratory syndrome coronavirus 2 (SARS‑CoV‑2) has led to an increased number of cases of morbidity and mortality [1]. The viral infection is very common in cases with compromised immunity like in patients of solid organ transplant (SOT) and the clinical presentations of these viral infections are usually atypical in this population often with two or more infectious processes presenting simultaneously [2,3]. There are various reports of COVID-19 in SOT cases. The weak immunity also makes these patients susceptible to other bacterial infections like *Mycobacterium tuberculosis*. The management of such cases with concomitant infections of a virus and bacteria is a formidable task. The situation becomes even more challenging when the health facilities are oversaturated due to COVID-19 [3]. There were multiple issues due to the pandemic like containment zones and lockdowns which adversely affected the timely presentation of cases in the outpatient department (OPD) [4]. This delay in management had a direct impact on the desired outcomes [4].

In this present case, the author highlights the management of a case of pulmonary tuberculosis (TB) with tuberculous right wrist tenosynovitis, hepatitis C, and mild COVID-19 in a kidney transplant Indian male. There was a history of repeated episodes of graft dysfunction and the situation became difficult with concomitant infections like COVID-19, hepatitis C, pulmonary and extrapulmonary TB. After detailed literature searches, it is correct to mention that this is the world’s first such case.

## Case presentation

On March 2021, a 24-year-old Indian male, a renal allograft recipient belonging to a low-income family, came as a referred case with chief complaints of a cough with yellow colored expectoration for two weeks, pale yellow colored discharge from the right wrist with right upper limb weakness for one week, and evening rise of high-grade fever for one week.

He was a non-alcoholic with no history of substance abuse. Further, there was no history of headache, nausea, rash, or any trauma. Besides, there was no history of TB, rheumatoid arthritis, or COVID-19 in him or his family. And he was a non-immigrant student with no history of imprisonment, unemployment, or contact with commercial sex workers or drug dealers. Also, there was no history of vaccination against COVID-19. The rest of his detailed history is mentioned in Table 1.

**Table 1:** Details of past history of the patient

Year	History
September, 2014	He received a renal allograft due to a left solitary kidney with vesicoureteral reflux (VUR). The transplant was done at a private hospital with the father as the donor. He was on tacrolimus (Tac)/mycophenolate mofetil/wysolone based immune suppression with baseline serum creatinine levels of 1.0-1.1 mg/dl.
May, 2015	He had an asymptomatic rise in serum creatinine levels (1.8 mg/dl) with low Tac levels (2.2 ng/mL). A graft biopsy was suggestive of acute T cell rejection (ACR1a) with antibody-mediated rejection (AMR) which was treated with injection methylprednisolone, plasma exchange, and bortezomib with serum creatinine settled to 1.4 mg/dl.
August, 2015	He again had an asymptomatic rise in serum creatinine levels (1.8 mg/dl) and a renal biopsy was suggestive of chronic interstitial inflammation with active tubulitis with interstitial fibrosis and tubular atrophy (IFTA) of 40% and was given antibiotic therapy.
November, 2015	He was admitted with urinary tract infection, graft dysfunction with serum creatinine levels (3.3 mg/dl), and anemia. The serum creatinine levels (1.3 mg/dl) settled after antibiotic therapy. Cytomegalovirus (CMV) deoxyribonucleic acid (DNA) polymerase chain reaction (PCR) showed 738 copies/ml and parvovirus DNA PCR was negative. During this admission, he was found to be HBsAg positive. And he was started on tablet entecavir 0.5 mg alternate days.
March, 2016	He was admitted due to complaints of high-grade fever, three episodes of vomiting, and four episodes of loose motion for two days. On investigation, it was attributed to graft dysfunction with serum creatinine levels increased to 3.8 mg/dl, urine routine and microscopy were suggestive of protein 2+, RBC 40-50/HPF, and WBC 40-42/HPF. Urine culture and sensitivity (C/S) were negative on multiple occasions and thus he was treated with IV antibiotics for culture-negative urinary tract infection (UTI). After three weeks of IV antibiotics, the serum creatinine levels were 3.2 mg/dl. A repeat graft biopsy was suggestive of ACR1a. His serum creatinine levels reduced to 2.2 mg/dl after four days of pulses of methylprednisolone.
April, 2016	Serum creatinine levels continued to raise thereafter and another graft biopsy was done which was suggestive of breakpoint cluster region protein (BCR) with AMR C4d+, IFTA grade 2, and acute tubular injury (ATI). Post discussion with a team of a pathologist, infectious diseases experts, and a nephrologist further immune suppression was not done due to multiple viral infections. His Tac levels were adequate at 5.8 ng/mL with a dose of 6.5 mg/day. And he was on tablet wysolone 7.5 mg once a day and tablet mycophenolate 720 mg twice daily. On his request, he was discharged to be followed up in the OPD at a serum creatinine level of 2.5 mg/dl.
September, 2016	He was admitted again with a lower respiratory tract infection (LRTI) and *Escherichia coli *positive UTI which was treated with antibiotics. He also acquired CMV infections and was given oral valganciclovir, 450 mg twice daily and tablet mycophenolate was withheld. He was discharged to be followed up in the OPD at a serum creatinine level of 2.2 mg/dl.
July, 2017	The patient was admitted again due to complaints of high-grade fever with dysuria. A urine C/S was suggestive of *Escherichia coli* positive UTI with serum creatinine levels of 5.2 mg/dl. He was given IV antibiotics for three weeks and a repeat urine C/S was sterile. His Tac levels were adequate at 5.3 ng/mL. USG-KUB showed 40 cc post-void residual (PVR) volume and uroflowmetry studies were suggestive of 100 cc PVR volume. Micturating cysto-urethrogram (or MCU) was not done in view of the UTI and the patient became stable with no major complaints and a serum creatinine level of 2.3 mg/dl.
October, 2017	He was again admitted with fever, graft dysfunction, and leucocyturia and was managed as graft pyelonephritis. During admission, he developed generalized tonic-clonic seizures due to a metabolic cause and was managed with anti-epileptics. His MRI brain and cerebrospinal fluid examination were unremarkable and a CT head and neck were suggestive of mastoiditis with maxillary sinusitis. He was managed conservatively and the swab for fungal and bacterial elements was sterile. But he developed Herpes zoster pancytopenia. A detailed evaluation after stopping all the drugs revealed a probable cause of bone marrow suppression. Further, a detailed evaluation for CMV, vitamin b12 levels, folate deficiency, parvovirus PCR, and bone marrow examination was done. His reticulocyte counts were normal. His peripheral blood smear revealed macroovalocytes which were managed with IV vitamin b12 and his anemia improved. His bone marrow examination was reported normal. Moreover, his tablet mycophenolate was stopped due to anemia and herpes zoster. He was discharged to be followed up in the OPD at a serum creatinine level of 2.7 mg/dl.
June, 2019	He came with complaints of swelling and pain in the right wrist joint for three days. A radiograph of the right hand with the forearm was normal. USG right wrist joint was suggestive of inflammatory tenosynovitis. These findings were confirmed on an MRI- of the forearm and hand which was indicative of diffuse flexor tenosynovitis extending from the forearm to the metacarpals. An orthopedician advised non-steroidal anti-inflammatory drugs (NSAIDs) and hot fomentation. The patient also had paresthesia around the distribution of C6 nerve roots. A nerve conduction study was done which was normal. Post NSAID use his paresthesia and tenosynovitis improved. His serum creatinine level was 2.0 mg/dl.

On examination, his vitals were temperature 101 degrees Fahrenheit, pulse 90 per minute, blood pressure 122/80 mmHg, oxygen saturation (SpO2) 95% on room air.

**Figure 1:**
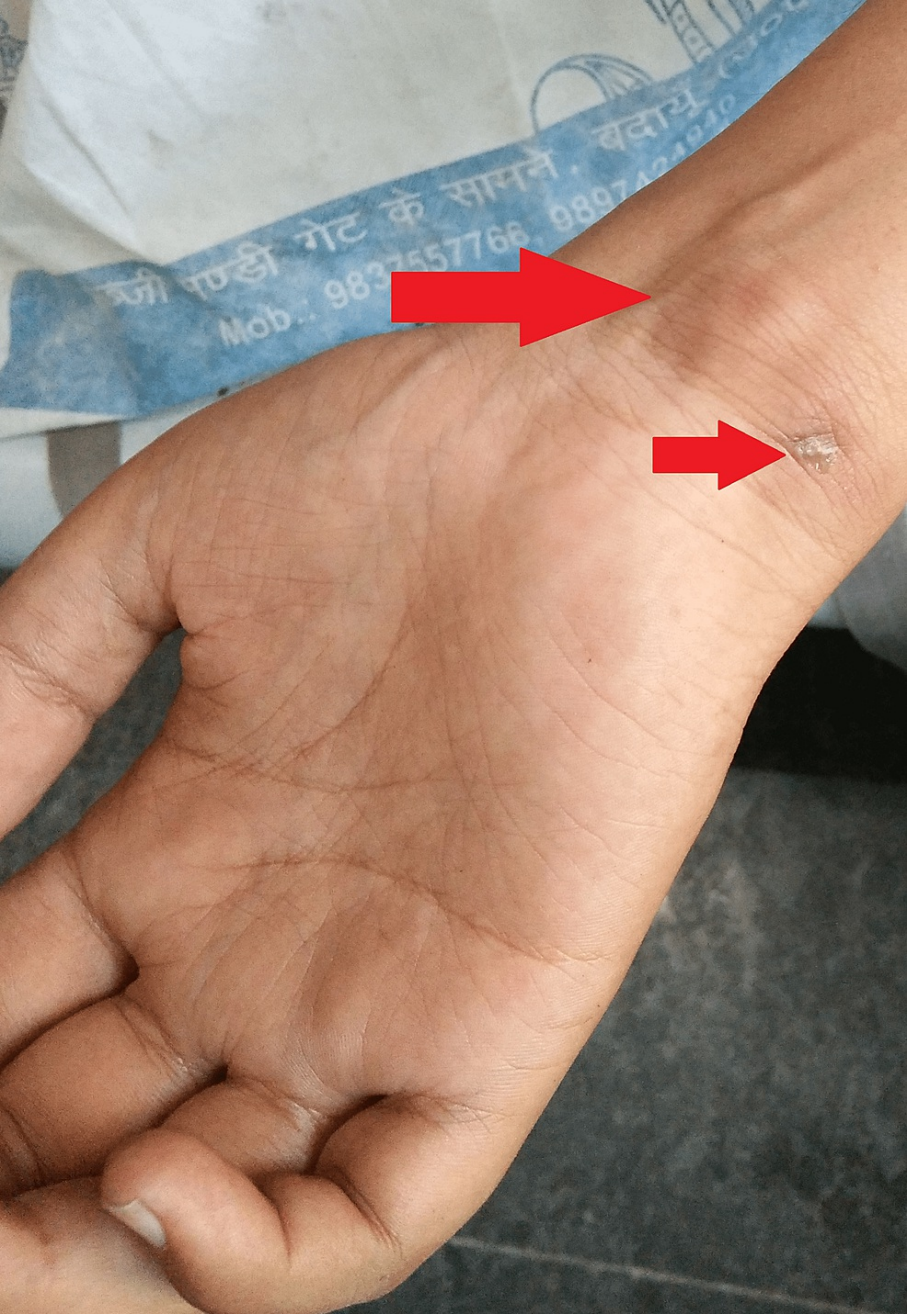
Right wrist showing a soft, non-pulsatile swelling and a discharging sinus with diffuse edema on the volar aspect

Local examination of the wrist revealed a soft, non-pulsatile swelling with tenderness and a discharging sinus with diffuse edema on the volar aspect (Figure 1). Kanavel’s cardinal signs were positive. Neurovascular examination of both extremities was normal. Systemic examination was unremarkable.

**Figure 2:**
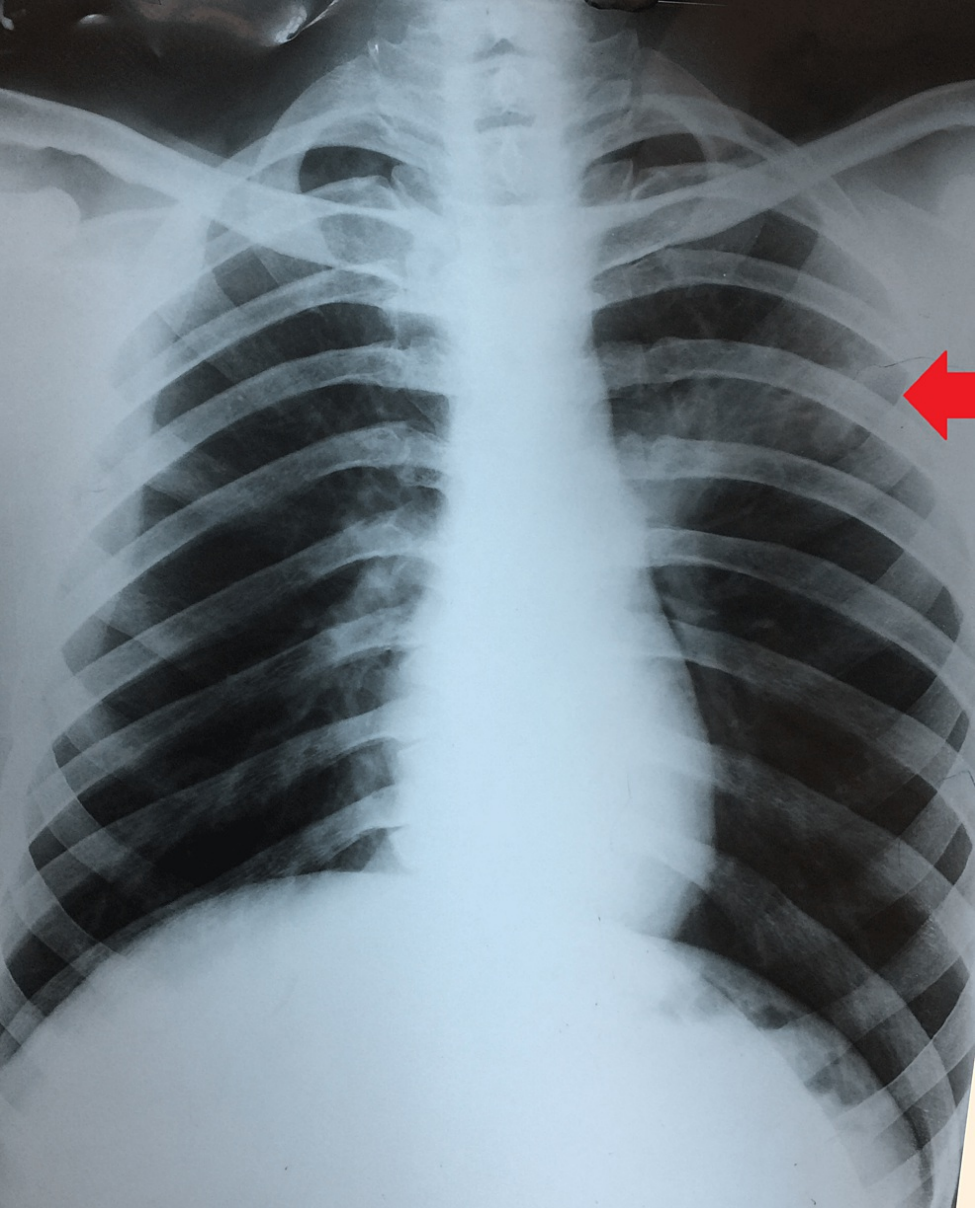
Chest radiograph (P-A view) showing left upper lobe consolidation P-A: Postero-anterior

He was provisionally diagnosed as a case of TB or COVID-19 and the same was confirmed with his sputum for acid-fast bacillus was positive for *M. tuberculosis* and the same was confirmed on cartridge-based nucleic acid amplification test (CBNAAT) with *M. tuberculosis* (low) sensitive to rifampicin. One more sample was sent for line probe assay and it was also suggestive of *M. tuberculosis* detected with no resistance to isoniazid or rifampicin. Further, a drug susceptibility test was not suggestive of any resistance to second-line anti-tubercular drugs. An on-spot rapid antigen test for SARS-CoV-2 was positive. A detailed laboratory work-up revealed hemoglobin of 9.0 g/dL, a raised erythrocyte sedimentation rate of 60 mm/hour, a serum creatinine of 1.8 mg/dl, and a reactive hepatitis C virus antibody test on enzyme-linked immunosorbent assay (ELISA) and it was confirmed on polymerase chain reaction (PCR) for viral RNA. HIV I and II were non-reactive. His chest radiograph (postero-anterior [P-A] view) was suggestive of left upper lobe consolidation (Figure 2). An electrocardiogram (EKG) was normal with QTc 360 ms.

A wrist X-ray was performed, which revealed an increase in soft tissue density. A USG-right upper limb indicated a collection of the size 2.1 cm X 1.3 cm X 0.8 cm in the medial aspect of the wrist around the intercarpal joint with a sinus opening into a tract on volar surface. There was synovial thickening around the intercarpal joint in the medial aspect. Radial, ulnar, and median nerves were normal. Furthermore, a nerve conduction velocity (NCV) test was done for upper limb weakness which was normal. The pus was sent for cytology which showed caseous granulomatous chronic inflammation compatible with TB and acid-fast gram stain positive and CBNAAT was positive for *M. tuberculosis *complex with no resistance to any drugs. Finally, a diagnosis of pulmonary TB with right wrist tenosynovitis, hepatitis C, and mild COVID-19 in a kidney transplant case was made and he was started on an anti-tubercular regimen with rifabutin (300 mg), isoniazid (300 mg), ethambutol (800 mg alternate day), and pyrazinamide (1500 mg alternate day). For COVID-19, he was given hydroxychloroquine 400 mg loading dose followed by 200 mg twice daily for a seven-day course, inhalational budesonide twice daily, vitamin B-complex once daily, vitamin C 500 mg three times a day, and betadine gargles twice daily. For hepatitis C, after consultation with a gastroenterologist, an oral fixed-dose combination of sofosbuvir (400 mg) and velpatasvir (100 mg) was started. Other medications which were advised after a careful evaluation of drug interaction at the discharge were capsule tacrolimus (3 mg in the morning and 1.5 mg at night), tablet Wysolone 15 mg for one week with tapering of dose, tablet levetiracetam 500 mg twice a day, and tablet vitamin B6 40 mg once a day. At his request, this patient was discharged to be followed up in the OPD. However, he was lost to follow-up.

## Discussion

Patients with renal transplants are vulnerable to various infections like TB, COVID-19, and hepatitis C [2]. TB could be pulmonary or extrapulmonary [4]. Extrapulmonary TB constitutes about 14% of all TB cases [5]. Of these, musculoskeletal system involvement is seen in about 1%-3% of cases, of which the hand as the main site of infection represents only 1% of cases [5,6]. Extrapulmonary TB has been reported at multiple sites beyond the lungs and could present as infectious tenosynovitis [5]. Tenosynovitis could be inflammatory, non-inflammatory, and infectious [5]. The exact incidence of infectious tenosynovitis is not known [5]. However, it is widely reported in the flexor tendons as compared to extensor counterparts [7]. And it is commonly caused by *Staphylococcus aureus* and less commonly by *M. tuberculosis* [5,7]. In this present case, the patient presented two years back with inflammatory tenosynovitis, and the same was managed with non-steroidal anti-inflammatory drugs (NSAIDs) and hot fomentation, but the development of infectious tenosynovitis in the same patient is a very rare entity.

Hepatitis C virus infection usually affects about 5%-15% of kidney transplant recipients in the developed world which is about 10 times higher than in the general population and is complicated with worse graft and patient survival [8]. If detected after the transplant it is very difficult to treat because of the poor efficacy, intolerable side effects, and risk of acute rejection associated with interferon-α (IFN) and ribavirin use [8].

COVID-19 is also reported in kidney transplant cases and immunosuppressive therapy including antilymphocyte therapy, a calcineurin inhibitor (e.g., Tac), high-dose corticosteroids, and mycophenolate mofetil may influence health status making patients more susceptible to COVID-19 [9]. Further, the adjustment of immunosuppressants and drugs for COVID-19 treatment to overcome drug interactions is a challenging task [9].

The author herein presented a post renal transplant patient who was simultaneously diagnosed as pulmonary TB with tuberculous right wrist tenosynovitis, COVID-19, and hepatitis C which is very rare and has never been reported in the literature. Cases of COVID-19 and TB in patients with a renal transplant are available in the literature and the cases of hepatitis C in post renal transplant patients are also available but concomitant presentations of all three infections have never been reported.

## Conclusions

The present case is a rare case where multiple infections due to viruses and bacteria in an immunosuppressed patient were diagnosed. A remarkable history with multiple admissions also makes this case interesting. Incidence of TB in post-transplant cases is very high and the same was found in this case. This patient also had a history of non-inflammatory tenosynovitis and later he was diagnosed with infectious tenosynovitis which is also very rare. To conclude, this case highlights the management of three infections and emphasizes the need for a very high degree of suspicion to rule-out various other infections in immunosuppressed patients.
